# The Influence of Kinematic Alignment on Patellofemoral Joint Biomechanics in Total Knee Arthroplasty

**DOI:** 10.3390/jcm13226894

**Published:** 2024-11-16

**Authors:** Johanna-Maria Simon, Leandra Bauer, Christoph Thorwächter, Matthias Woiczinski, Florian Simon, Peter E. Müller, Boris M. Holzapfel, Thomas R. Niethammer

**Affiliations:** 1 Department of Orthopaedics and Trauma Surgery, Musculoskeletal University Center Munich (MUM), LMU University Hospital, LMU Munich, 81377 Munich, Germany; 2 Experimental Orthopaedics, University Hospital Jena, Campus Eisenberg, Waldkliniken Eisenberg, 07607 Eisenberg, Germany; 3 Department of Otorhinolaryngology, LMU University Hospital, LMU Munich, 81377 Munich, Germany

**Keywords:** kinematic alignment, total knee arthroplasty, patellofemoral joint, biomechanics, quadriceps load

## Abstract

**Background**: Anterior knee pain is a prevalent issue post total knee arthroplasty, often necessitating revision surgery. Various factors contribute to this complication, including patellar maltracking and excessive patellofemoral load. Kinematic alignment has emerged as an alternative, showing promising outcomes in clinical studies. However, its impact on patellofemoral biomechanics needs to be more adequately understood. This study compared the effects of kinematically versus mechanically aligned total knee arthroplasty on patellofemoral joint biomechanics. **Methods**: Eight fresh-frozen human knee specimens underwent biomechanical testing in a knee rig setup, performing an active weight-loaded knee joint flexion of 30–130°. After the testing of native kinematics, kinematically and mechanically aligned total knee arthroplasty was performed using a medial pivot implant design without patellar resurfacing. Quadriceps force, retropatellar peak pressure and the retropatellar contact area were measured during knee flexion using a patellar pressure-sensitive film. Patella kinematics (shift and tilt) was tracked using an optoelectrical measurement system. Functional regressions were used to determine the influence of the alignment on the kinematics and loading of the knee joint. **Results**: Kinematically aligned total knee arthroplasty resulted in reduced quadriceps force during knee flexion compared to mechanically aligned total knee arthroplasty. Retropatellar peak pressure, retropatellar contact area and patella kinematics did not vary between the alignments. **Conclusions**: Kinematic alignment offers potential benefits in reducing quadriceps force during knee flexion, which may mitigate anterior knee pain risk. Further research is needed to elucidate its effects in varying anatomical conditions and alignment strategies.

## 1. Introduction

Up to 10% of patients following total knee arthroplasty (TKA) suffer from anterior knee pain [1]. Anterior knee pain is therefore the most common cause of persistent problems after TKA and of revision surgery in the early postoperative phase [2].

Several studies show an apparent correlation between the prevalence of anterior knee pain, abnormal patellar tracking, and higher patellofemoral contact pressure [3,4,5,6]. Likewise, functional reasons such as an imbalance of the quadriceps muscle are potential causes of anterior knee pain [7]. An incorrect rotation of the femoral component is discussed as the most common cause of patellar complications, whereupon, according to Akagi et al., a 3 to 5° external rotation of the femoral component shows improved patellar tracking patterns [8]. Computer modelling by Thompson et al. demonstrated a substantial influence of femoral component rotation on patella pressure distribution as well as on quadriceps and collateral ligament forces [9]. Moreover, external femoral component rotation also led to less quadriceps demand.

Increased patellofemoral loads have also been attributed to the overstuffing of the patellofemoral joint, i.e., due to oversized femoral components or a more anterior component positioning [10].

However, Insall et al. suppose that patellar kinematics also are mainly influenced by the joint line orientation, q angle and overall limb alignment [11,12].

In mechanical alignment (MA), the tibiofemoral joint line cut is carried out perpendicular to the mechanical leg axis and the femoral component is implanted following a three-degree external rotation with reference to the posterior condyle axis, independent of the individual anatomy, and often results in the need of ligament release [13]. However, the hip–knee–ankle (HKA) angle and the tibiofemoral joint line obliquity, as well as tibial slope and trochlear angles, are known to have high interindividual variability [14,15,16]. Hence, MA TKA may lead to significant changes in the native coronal alignment of the patient [17], whereas several studies have shown that small modifications in the alignment of the femoral component result in significant changes in patella tracking [18,19]. Therefore, it may be reasonable to restore normal patellofemoral and tibiofemoral kinematics in order to reduce the risk of anterior knee pain after TKA as changes in the tibiofemoral joint line can be attributed to deviated patellofemoral joint orientation due to standardized femoral component design.

This can be attributed to the concept of kinematic alignment (KA), which attempts to restore the patient’s constitutional prearthritic alignment, taking into account the individual HKA angle and joint line obliquity by merely a caliper-verified resection of the remaining cartilage and bone, which is going to be replaced by the implant [20]. This way, KA TKA is able to prevent ligament laxity and the need of ligament release, respectively.

According to current studies, KA has resulted in improved clinical outcomes and a more physiological feeling of the knee [21,22,23]. Simultaneously, long-term implant survival and complication rates of KA TKA were similar to those of MA TKA [24,25]. However, specific information regarding the potential benefit of KA TKA with the prevalence of anterior knee pain is lacking.

Despite this, there also is a relevant lack of data regarding the influence of KA TKA on patellofemoral joint biomechanics, especially since KA TKA restores individual joint line orientation and therefore might lead to variable deviation of the patellofemoral joint geometry by the use of standardized implants, primarily designed for MA, which can be assessed by in vitro measurements of patellar kinematics and patellofemoral loads using an established knee rig and human cadaver specimen [5,26]. These data could subsequently be used to gain a better understanding of the origin of patellar pressure loads and the effects of different tibiofemoral alignment strategies, especially of the clinically promising kinematic alignment, and might contribute to the development of individual alignment options respecting individual leg axis alterations.

The purpose of this study was to compare the influence of KA TKA versus MA TKA on patellofemoral joint biomechanics in relation to the native situation (NS). Here, we hypothesized that KA TKA showed a closer reconstruction of native aspects in patellar kinematics and patellofemoral load patterns due to the attempt of restoring individual patellofemoral joint geometry.

## 2. Materials and Methods

### 2.1. Specimen and Implantation

Ethical approval was given by the Ethics Committee of the University of Munich, #20-829. Eight fresh-frozen human specimens were used for biomechanical testing. The specimens consisted of four males and four females with an average age of 80 (±6) years. They presented with a neutral HKA angle and a distal apex joint line obliquity equivalent to Coronal Plane Alignment of the Knee classification (CPAK) type II, which was revealed to be the most common phenotype in a healthy population and comprises a neutral HKA angle and a joint line dipping from the lateral to the medial side [27].

Prior to the experiment, the specimens were thawed at room temperature for 24 h and prepared by removing the skin and unnecessary tissue. Medical suture material was used to fix the tendons of the quadriceps femoris, biceps femoris, and semitendinosus muscles in finger clamps. The length of the tibia and femur were shortened to 22 cm and 20 cm respectively, measured from the epicondylar axis. To ensure stability during the experimental setup, the fibular head was attached to the tibia with a cortical screw. The knee joint was clamped in the knee rig by embedding the ends of the tibia and femur in metal shells with epoxy resin [28,29,30]. To reduce the malalignment of the femoral bone within the metal cups, the posterior condyles of the femur were rotated internally/externally and aligned parallel to the hip flexion axis of the experimental setup in the transverse plane.

For TKA, the GMK Sphere (Medacta International, Castel San Pietro, Switzerland) total knee system was applied, using a medial pivot/medial-stabilized (MS) polyethylene insert. Although the GMK system comprises manual instruments specifically for MA TKA as well as KA TKA, the implants used are solely designed for use within MA TKA. All experiments were started with KA followed by MA. The posterior cruciate ligament was retained, independent of the alignment technique. The patella remained unsurfaced. TKA was carried out by two experienced surgeons according to the standard procedure as defined by the instructions manual of the implants.

At first, the femoral distal cut reference was marked using two pins with a standard 6° valgus correction measured from the anatomical axis (as indicated by the manual for MA) to guarantee a correct femoral distal cut for MA later on. Next, with regard to the technique of caliper-verified unrestricted KA, a distal cut reference labelled “worn” with contact with both femoral condyles was set after a resection of remaining cartilage due to inconsistent amounts of osteoarthritis within the specimen. The distal femoral cuts (6 mm thickness) were preserved to allow us to switch to the MA technique afterwards. Femoral component rotation was aligned to the posterior condyle axis. The tibial cut was performed matching the coronal proximal tibial joint and the medial tibial slope.

Thereafter, MA TKA was performed using the same TKA system and implants. For this purpose, the distal femoral resected cuts were reattached to the femoral condyles using Kirschner wires and the distal cutting block was fixed using the premarked pin holes. Considering the 1.27 mm bone loss due to the saw blade’s thickness, the distal cutting block was moved 2 mm proximal (4 mm in one case only) to allow for sufficient resection amounts. However, in MA TKA, the distal femoral cut is performed with a fixed 6° valgus correction to the anatomical axis of the femur to result in a perpendicular joint line orientation. Hence, particularly in common CPAK types I and II, the distal femoral cut is performed in reference to the medial femoral condyle, resulting in a narrowed lateral distal femoral cut and therefore preventing the overall proximalization of the tibiofemoral joint line. Femoral component rotation was kept identical to KA to avoid potential bias by modifying femoral component rotation, and therefore, anterior/posterior and chamfer cuts remained the same. The tibial cut was performed using intramedullary alignment instruments with a standard 3° tibial slope. In addition, the cutting block was adjusted for a sufficient tibial resection amount, facilitating implant stability while avoiding substantial loss of bone stock.

For both KA TKA and MA TKA, the same insert size (10 mm thickness) showed sufficient ligament stability in flexion and extension.

### 2.2. Biomechanical Setup

**Figure 1:**
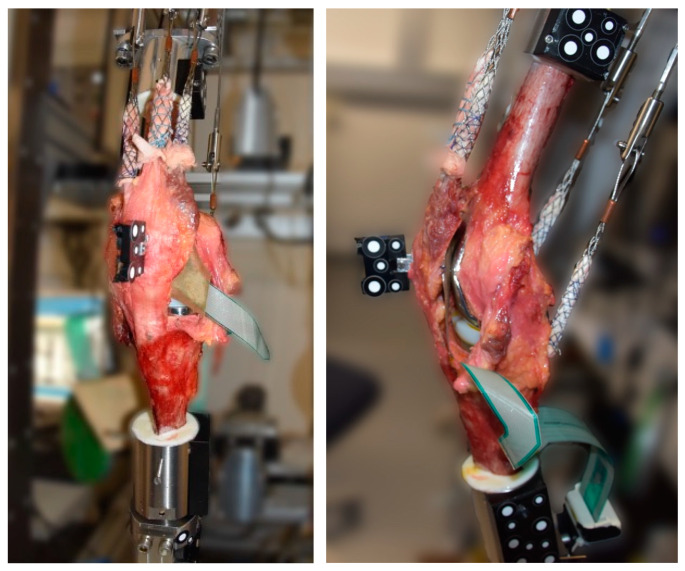
Experimental setup with tendons attached to finger traps, optoelectrical markers at the femur, tibia and patella and pressure-sensitive film within the patellofemoral joint.

The specimens were tested in the established knee rig [29,31]. This setup has six degrees of freedom and can perform an active, weight-loaded knee flexion of 30–130° with a constant ground reaction force (GRF) of 50 N. The hip joint is supported in the vertical axis by means of a linear guide (1st degree of freedom), thereby enabling a translation to the ankle joint. Furthermore, the femur is capable of flexion/extension rotation (2nd degree of freedom) at the hip joint. The ankle joint is freely supported in translation (3rd degree of freedom), which enables the equalization of the joint in the medio-lateral plane. The fourth and fifth degrees of freedom, namely abduction/adduction and flexion/extension rotation, are released via a universal joint. The axial bearing allows for the free rotation of the tibia in both the internal and external directions, representing the sixth degrees of freedom [28,29,30]. The constant GRF was controlled by the rectus femoris muscle using a sensor (8417-6002 Burster, Gernsbach, Germany). The activity of the vastus medialis, vastus lateralis, semitendinosus and biceps femoris muscles was simulated using 2 kg weights attached to the tendons. A self-programmed LabView program controlled the movement in real time (version 8.6, National Instruments, Austin, TX, USA). An optoelectrical measurement system (ARAMIS 3D Camera 2.3M, GOM GmbH, Braunschweig, Germany) was used to record the movement of the specimen using reflective markers on the femoral and tibial heads and on the patella (Figure 1). The kinematics of the patellofemoral joint (patella shift and tilt) were calculated using the movement data from the optoelectronic measuring system. The method used has been demonstrated to be effective and has been described in detail previously [32,33]. For the calculation of the local coordinate system, several landmarks of the knee joint were recorded in the native situation and used over the entire trial of a specimen.

Retropatellar pressure distribution was measured using a thin pressure-sensitive film (K-Scan 4000, Tekscan Inc., Boston, MA, USA) attached to the retropatellar surface [34,35].

At first, the NS was assessed after preparing the knee specimen as described above (joint capsula opened, pressure-sensitive film attached to the patella). Thereafter, measurements of KA TKA and MA TKA were performed, with the knee rig performing a deep knee bend as described above.

### 2.3. Data Analysis and Statistics

The data from the optoelectrical measuring system were synchronized and interpolated with the flexion angle and recorded by the knee rig program. Further data analysis was carried out using a self-programmed MATLAB script (MathWorks Inc., Natick, MA, USA). Peak pressure was calculated by averaging the maximum value over a window of the eight surrounding values in order to circumvent artefacts (in accordance with the methodology proposed by [36]). The contact area was determined by the number of pixels that exceeded a value of 0 MPa.

The results shown here are the means across eight knee joints with a 95% confidence interval (CI). R Studio software (R version 4.3.1) was used for the statistical analysis. Functional regressions (pffr) were calculated using the refund (v 0.1-35) library [37]. The NS was treated as a reference variable and the effect of MA and KA on the respective target variables (quadriceps force, peak pressure, contact area, shift and tilt) was determined over the flexion cycle. The results therefore show the fixed influence of the NS in the results of the intercept.

## 3. Results

### 3.1. Patellofemoral Load Situation

**Figure 2:**
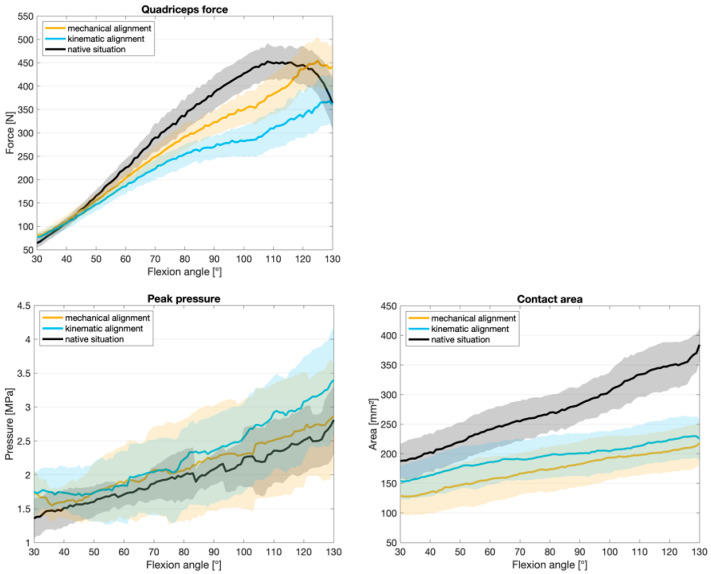
Mean values with 95% confidence interval for quadriceps force, retropatellar peak pressure and retropatellar contact area.

**Figure 3:**
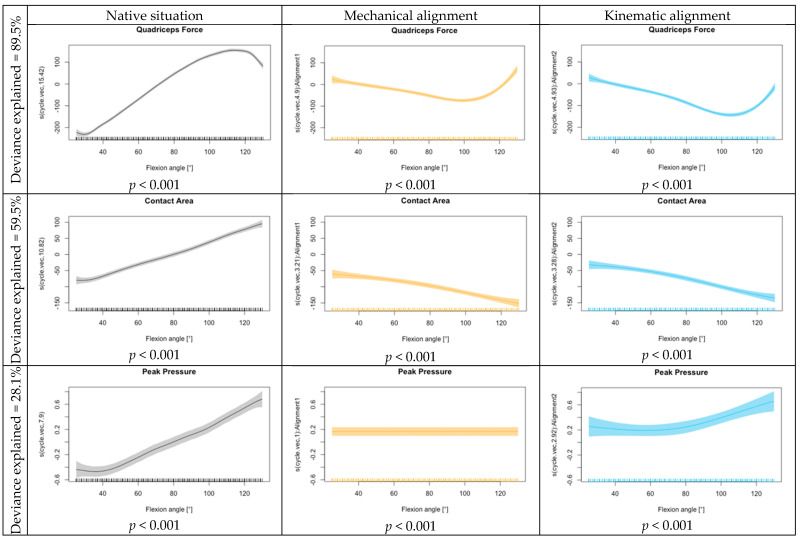
Functional regression quadriceps force, contact area and peak pressure for the intercept (native situation), mechanical alignment and kinematic alignment.

A comparison of KA, MA, and the NS for quadriceps force, retropatellar peak pressure and retropatellar contact area is shown in Figure 2. The lowest quadriceps force was required for the flexion of the knee joint from 70° flexion onwards when a KA TKA was implanted. With MA TKA, the quadriceps force was slightly higher from 60° onwards but still lower than for native knees. With KA, significant results are shown in the functional regression across the entire knee flexion. With MA, the quadriceps force is less influenced at the beginning. The KA showed an increase in the influence on peak pressure at lower flexion angles. Meanwhile, the effect for MA remained constant over the flexion cycle. Any arthroplasty resulted in a reduction in the retropatellar contact area compared to the NS, regardless of the alignment technique. In the functional regression, a slightly increased effect on the contact area was shown for MA. The progression over the knee bend did not differ between MA and KA. Both alignments have a significant influence on the contact area compared to the NS (*p* < 0.001) (see Figure 3).

### 3.2. Patellofemoral Kinematics

**Figure 4:**
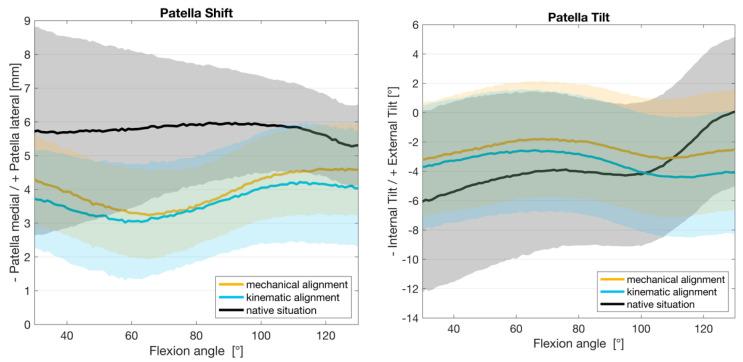
Kinematic results for patella shift and tilt with mean and 95% confidence interval.

Figure 4 displays the kinematics of the patellofemoral joint including mean values and 95% confidence intervals for all eight knee joints. In its natural state, the patella exhibited minimal shift movement, with a slight tendency towards the medial side in the deepest flexion. This movement was altered following TKA. The knee joints with KA and MA showed a similar pattern throughout the flexion cycle. At the onset of 30° flexion, the patella was more medialized. It continued to move medially up to a mean flexion of 60° before returning to a lateral position with higher flexion (up to 130°). The MA showed a slightly lower influence on the shift at the beginning and end of the flexion movement compared to the KA.

**Figure 5:**
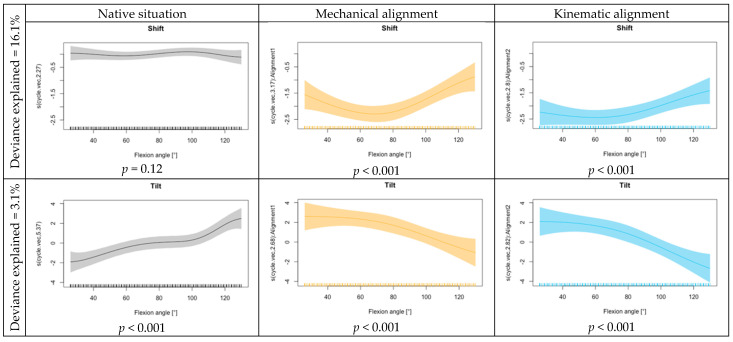
Functional regression for shift and tilt for the intercept (native situation), mechanical alignment and kinematic alignment.

During tilt movement, the patella exhibited a comparable pattern up to 80° flexion in both native and TKA situations (MA and KA). The study found that there was an external tilt of the patella in its natural state, which changed to a slight internal tilt with flattening in higher flexion after TKA. The functional regression also shows a similar effect across the squat movement for the alignment options regarding tilt movement (see Figure 5).

## 4. Discussion

The main findings of this study were a greater reduction in quadriceps force for KA TKA compared to MA TKA. These results account for a lower amount of required muscle effort to extend the knee, which might have a positive impact on the functional outcome after TKA. This assumption can be supported by a study of Mizner et al., who examined functional measures of patients, including quadriceps strength, undergoing unilateral TKA pre- and postoperatively over a period of 6 months. They detected a high correlation between regaining quadriceps strength and functional outcome postoperatively via functional tests and knee function questionnaires [38]. Moreover, Stevens et al. observed that patients with osteoarthritis of the knee show quadriceps weakness caused by arthrogenous muscle inhibition, which persists after TKA in spite of pain relief [39]. Hence, although the reduction in quadriceps load does not correspond to the reconstruction of the in vitro NS, it may be beneficial in terms of higher functional outcomes after KA TKA.

In addition, already in 1998, Pagnano et al. showed that the overloading of the quadriceps muscle, due to flexion instability after TKA, correlates with a higher incidence of anterior knee pain [40]. This empowers the assumption that a reduced quadriceps force also might have a positive impact on the occurrence of postoperative anterior knee pain as the insertions of the extensor apparatus are expected to be a main source for pain sensation within the anterior knee [41,42,43]. In summary, the reduced quadriceps force, as seen after KA TKA, might be a meaningful contributor to a better functional outcome and reduction in anterior knee pain.

The reduction in quadriceps force after KA TKA compared to MA TKA can be explained by our findings of increased lateral femoral rollback during flexion movement after KA TKA in another in vitro study investigating femorotibial kinematics comparing KA vs. MA TKA [44]. This is based on the knowledge of native patellofemoral joint kinematics, which respects the function of the patella as a fulcrum to increase the moment arm of the quadriceps muscle, increasing the muscle’s effectiveness in knee extension, whereby the positioning of the tibia in relation to the femur plays an important role [45]. On this basis, increased lateral femoral rollback in higher flexion rates after KA TKA leads to a more anterior position of the lateral tibia, which contributes to an optimized moment arm of the quadriceps muscle and significantly reduced quadriceps force.

The phenomenon of a significant reduction in quadriceps force after TKA compared to the NS cannot easily be explained. Evaluating patellofemoral contact patterns before and after TKA using the same established Munich knee rig, Steinbrück et al. observed similar amounts of quadriceps force but less internal tibia rotation and higher retropatellar peak pressure after MA TKA [35]. However, they used a cruciate-retaining TKA implant (Aesculap Columbus CR), which illustrates a major distinction to our experimental setup with a medial pivot knee. Thus, the implant design, i.e., due to the femoral component radius or deepest saddle point of the insert, may have already led to a certain amount of the optimized moment arm of the quadriceps muscle.

Furthermore, the resection of the anterior cruciate ligament during TKA may also be a contributing factor to reduced quadriceps force after TKA compared to the NS since anterior cruciate deficiency in a native knee leads to an increase in the anterior translation of the tibia and therefore contributes to an optimized moment arm of the quadriceps muscle [46].

After TKA, independent of the alignment strategy, we can observe a reduction in the retropatellar contact area, expectedly. This is mainly due to a certain mismatch between individual retropatellar surface areas and the trochlear groove of the femoral component design used. However, the patella is responsible for the increase in knee extension forces by approximately 50% and serves to increase the surface area of force distribution [47]. Therefore, when performing TKA, an even and large-scale retropatellar contact area should be aimed for to avoid a rise in retropatellar peak pressure and its presumably associated anterior knee pain [3,4,5].

Currently, there are only two preexisting in vitro studies by the same group (Kim and Koh et al.) examining the patellofemoral joint after KA TKA [48,49]. Both studies consisted of human specimens comparing KA TKA vs. MA TKA, with subsequent measurement of kinematics and patella tracking patterns within a mainly passive knee rig. The results showed a more lateral position of the patella (at 90°) for MA TKA compared to KA TKA and the NS and similar patella tracking patterns to the native knee regarding KA TKA. MA TKA also contributed to a higher pressure load of the lateral patellofemoral joint compared to the NS and KA TKA.

In contrast, in our study, MA TKA and KA TKA both led to a medialization of the patella compared to the NS, especially at a 60–80° flexion angle, similar to findings by Steinbrück et al., which were independent of the mediolateral femoral component position [31]. The trochlear groove is mainly located laterally to the mid-plane of the condyles [50], with high variance in trochlear orientation [51]. Next to the significant alteration of trochlea orientation caused by the uniform design of the implants, a systematic medial error of the trochlear groove after TKA due to asymmetrical distal resection areas and the equal width of femoral component condyles, which lead to a medialization of the femoral component, is suspected [52,53]. Although the femoral component of the GMK Sphere provides a sulcus 2 mm lateral of the centre, this issue may not adequately be addressed enough.

The different angle of the tibiofemoral joint line due to the contrary alignment strategies did not lead to an increased medial patella shift after KA TKA. The lack of difference in patella shifting within our study comparing KA TKA vs. MA TKA might be explained by the circumstance that we referred to femoral component rotation according to the posterior condyle axis for both alignment strategies in order to avoid a potential incalculable bias in addition to the tibiofemoral joint line orientation.

Nevertheless, these findings support the need of a specific femoral component design for KA TKA incorporating a more lateral positioning of the trochlear groove compared to the MA TKA design [54].

Regarding this study, several limitations must be considered. First of all, the present study is an in vitro study with a small sample size due to the high effort and ethical aspect of using cadaver specimens, yet this is similar to other in vitro studies using a knee rig. It is important to note that there is no proprioception that could potentially influence human movement. Moreover, the present test configuration is inadequate for modelling all muscles with regard to cocontractions during an active knee bend. However, the use of cadaveric specimens in biomechanical testing remains a valuable and essential tool in the field [55].

On the basis of the high power of functional regression analysis, nearly all tested variables showed significant differences, whereupon the rate of explained deviance often remained low (i.e., patella shift/tilt) due to the distribution and sample size.

For this experiment, cadaver specimens with a healthy knee joint and straight leg axis were used to guarantee comparability. Since the KA technique especially leads to relevant alignment differences within varus and valgus legs and the severity of osteoarthritis also influences the implantation process, a relevant leg axis alteration and the presence of severe osteoarthritis might lead to an even higher impact on potential biomechanical and kinematic measurements. Additionally, the implantation technique used, regarding the retention of femoral component rotation during the transition from KA TKA to MA TKA, may have led to minor significant results and a negative effect on patellofemoral kinematics after MA TKA.

Finally, the mere existence of knee implants, designed for MA, limits the flexibility of patella positioning and may lead to restricted unphysiological patellofemoral guidance.

## 5. Conclusions

KA TKA facilitates the extension of the knee by reducing the quadriceps force needed, which may implicate a benefit of KA over MA when a knee arthroplasty is performed since a reduction in physical fatigue and anterior knee pain can be expected. Nonetheless, changes in retropatellar peak pressure and contact area as well as patella shift and tilt were independent of the alignment strategy used.

However, the lack of specific implants for KA and human specimens with relevant leg axis alterations (varus/valgus) limits the power of this in vitro study.

## Data Availability

The datasets used and/or analysed during the current study are available from the corresponding author on reasonable request.

## Abbreviations

TKA	total knee arthroplasty
KA	kinematic alignment
MA	mechanical alignment
NS	native situation
CPAK	coronal plane alignment of the knee
MS	medial-stabilized
GRF	ground reaction force
HKA	hip–knee–ankle
